# Poly(acrylic acid)-regulated Synthesis of Rod-Like Calcium Carbonate Nanoparticles for Inducing the Osteogenic Differentiation of MC3T3-E1 Cells

**DOI:** 10.3390/ijms17050639

**Published:** 2016-05-06

**Authors:** Wei Yang, Chenxue Yao, Zhengyang Cui, Dandan Luo, In-Seop Lee, Juming Yao, Cen Chen, Xiangdong Kong

**Affiliations:** 1 Bio-X Center, College of Life Sciences, Zhejiang Sci-Tech University, Hangzhou 310018, China; vivi-yangwei@hotmail.com (W.Y.); yaochenxue092@163.com (C.Y.); cuizy2015@126.com (Z.C.); dandanlaw@126.com (D.L.); chencen313@gmail.com (C.C.); 2 Institute of Natural Sciences, Yonsei University, Seoul 120-749, Korea; inseop@yonsei.ac.kr; 3 College of Materials and Textiles, Zhejiang Sci-Tech University, Hangzhou 310018, China; yaoj@zstu.edu.cn

**Keywords:** calcium carbonate, nanoparticles, rod-like, bone regeneration

## Abstract

Calcium carbonate, especially with nanostructure, has been considered as a good candidate material for bone regeneration due to its excellent biodegradability and osteoconductivity. In this study, rod-like calcium carbonate nanoparticles (Rod-CC NPs) with desired water dispersibility were achieved with the regulation of poly (acrylic acid). Characterization results revealed that the Rod-CC NPs had an average length of 240 nm, a width of 90 nm with an average aspect ratio of 2.60 and a negative ζ-potential of −22.25 ± 0.35 mV. The degradation study illustrated the nanoparticles degraded 23% at pH 7.4 and 45% at pH 5.6 in phosphate-buffered saline (PBS) solution within three months. When cultured with MC3T3-E1 cells, the Rod-CC NPs exhibited a positive effect on the proliferation of osteoblast cells. Alkaline phosphatase (ALP) activity assays together with the osteocalcin (OCN) and bone sialoprotein (BSP) expression observations demonstrated the nanoparticles could induce the differentiation of MC3T3-E1 cells. Our study developed well-dispersed rod-like calcium carbonate nanoparticles which have great potential to be used in bone regeneration.

## 1. Introduction

Various bone diseases such as bone fractures, osteoporosis, osteoarthritis, and bone cancers commonly present urgent clinical needs for bone regenerative materials [1]. Implant materials in the clinic can sometimes cause serious side effects such as inflammation and infection which create the necessity to develop new regenerative materials [2,3,4]. Among new kinds of materials, hydroxyapatite, which is the main ingredient of bone, has been widely used [5,6,7,8]. However, in contrast to calcium carbonate, the poor biodegradability of hydroxyapatite has limited its applications in biomedical fields [9,10,11]. Calcium carbonate has been considered to be an ideal bone repair material based on its excellent osteoconductivity and biodegradability [12,13,14,15].

Calcium carbonate in nano-scale size has attracted more attention in bone regeneration due to its flexibility in preparation, its ability to enhance cell attachment and proliferation and its high efficiency in carrying more drug or bioactive molecules [16,17,18,19]. Yu employed macro-, micro- and nano-scale hierarchical calcium carbonate scaffolds to repair bone defects and proved that the scaffold with nanostructured calcium carbonate enhanced the protein adsorption and accelerated its continuous degradation, thus providing high calcium for promoting bone growth [20]. Fujihara confirmed calcium carbonate nanoparticles’ dispersed polycaprolactone nano-fibers improved the attachment and proliferation of osteoblast cells [16]. Calcium carbonate nanoparticles with diverse sizes and shapes have been synthesized and their interactions with cells have been systematically studied [21,22]. According to early studies, rod-like nanoparticles have better interactions with proteins and cells to enhance cellular uptake and intracellular sorting [23,24,25]. However, not many reports have demonstrated the specific effects of rod-like calcium carbonate nanoparticles on osteoblast cells.

In the present study, rod-like calcium carbonate nanoparticles were synthesized with the regulation of poly(acrylic acid) (PAA). The physical-chemical properties, *in vitro* degradation and cytocompatibility of the prepared nanoparticles were investigated. Besides, the *in vitro* proliferation and differentiation assays on MC3T3-E1 cells were conducted to evaluate its effect as bone regenerative material.

## 2. Results and Discussion

### 2.1. Preparation and Characterization of Rod-Like Calcium Carbonate Nanoparticles (Rod-CC NPs)

**Figure 1:**
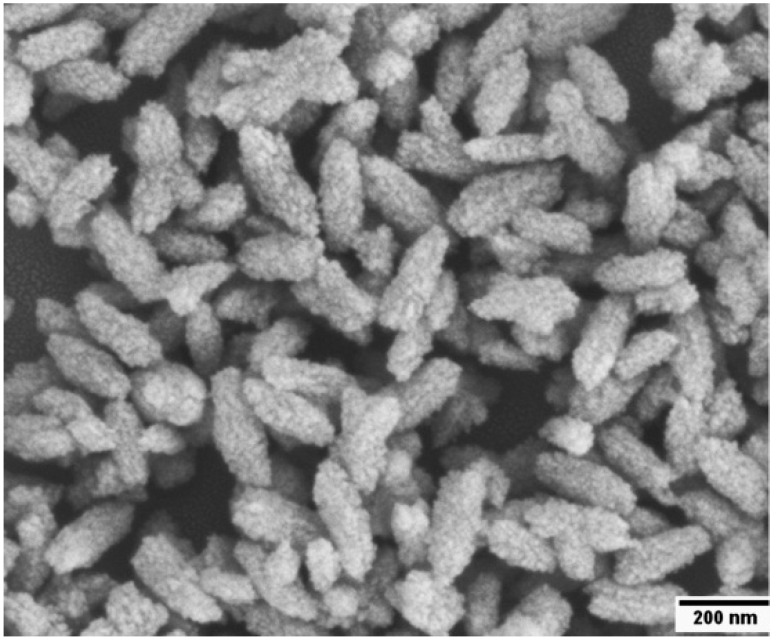
SEM image of the prepared rod-like calcium carbonate nanoparticles (Rod-CC NPs).

PAA was used to regulate the synthesis of calcium carbonate nanoparticles. The SEM (Figure 1) image shows that the prepared nanoparticles exhibited a uniform rod shape with an average length of 220 nm and a width of 85 nm, and the SEM image also displays the rough surfaces of Rod-CC NPs. The rough surface enabled Rod-CC NPs to load more drugs or bioactive molecules which is beneficial for its further applications.

**Figure 2:**
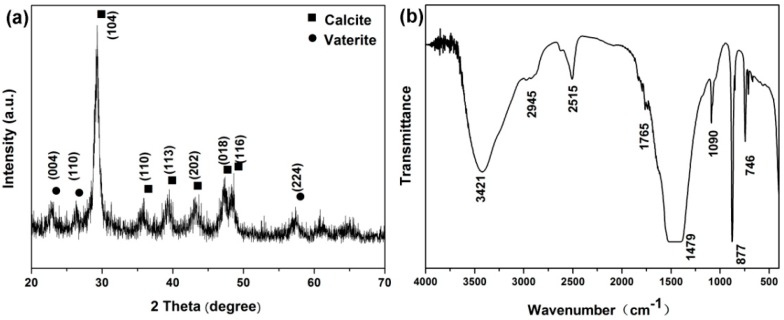
XRD patterns (**a**) and FTIR (**b**) spectrum of Rod-CC NPs.

The XRD pattern (Figure 2a) indicates that the nanoparticles were composed of a high crystallinity of calcite (JCPDS 47-1743), as shown by the sharp diffraction peaks, and a low vaterite (JCPDS 33-0268). The crystal phase of the obtained calcium carbonate products was also characterized by the FTIR spectrum. As shown in Figure 2b, the absorption peak located at 877 cm^−1^ was the characteristic peak of calcite, and the peaks at 745 and 1090 cm^−1^ were for vaterite.

**Figure 3:**
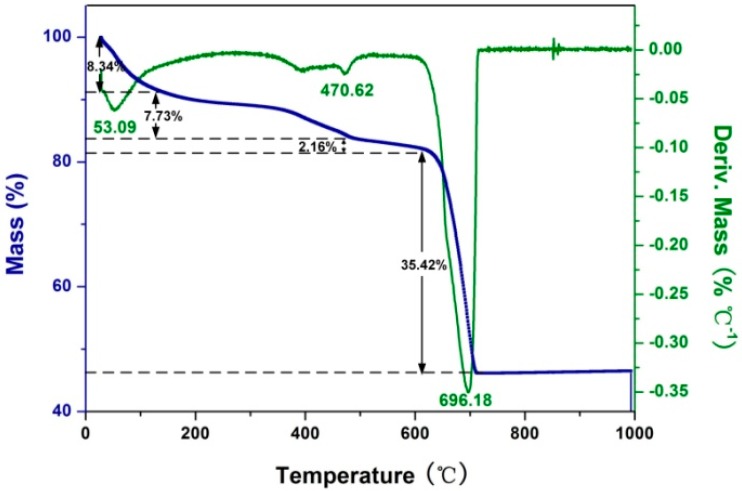
TGA and DTG curves of Rod-CC NPs.

TGA was employed to investigate the percentage of PAA that was involved in the precipitation of the mineral crystalline. Figure 3 shows the TGA and DTG curves of PAA-regulated Rod-CC NPs. The first decomposition between 25 and 123 °C with a maximum rate at 53 °C and weight loss of almost 8.34 wt % could be attributed to water loss. Additionally, the second stage, at 500 °C with a maximum decomposition rate at 470 °C and weight loss of about 7.73 wt %, was the degradation of PAA. The last curve was for the decomposition of calcium carbonate which occupied almost 84 wt % of the total Rod-CC NPs. Besides, the last stage consisted of the decomposition of vaterite (loss of 2.16 wt %) and calcite (loss of 35.42 wt %). The 7.73 wt % PAA involved in Rod-CC NPs played an important role in modifying the surface charge with its carboxyl which may contribute to its ζ-potential of −22.25 ± 0.35 mV.

**Figure 4:**
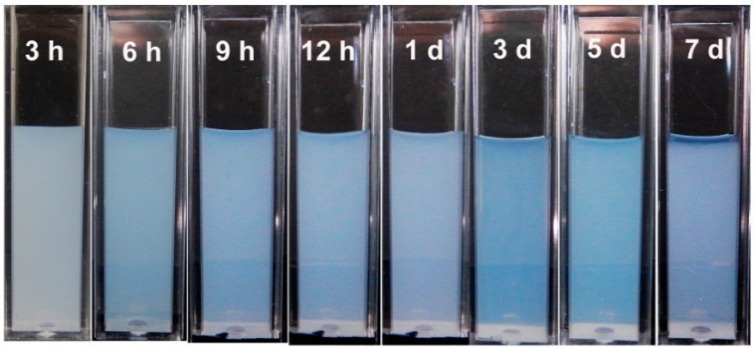
Photographs of Rod-CC NPs at a concentration of 1 mg/mL after standing still for 3, 6, 9, 12 h and 1, 3, 5, 7 day.

The aqueous dispersion and stability were also studied. As shown in Figure 4, the Rod-CC NPs at a concentration of 1 mg/mL dispersed very well with little sediment for at least one week, which implied its good aqueous dispersion and stability. Its desired aqueous stability could be attributed to the involved PAA which formed a negative charge layer, preventing the aggregation of Rod-CC NPs in aqueous solution [26].

**Figure 5:**
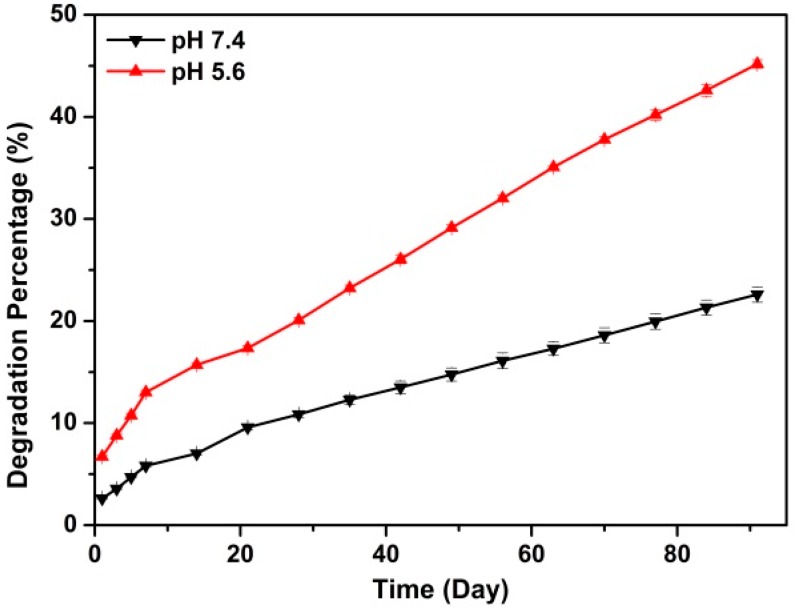
*In vitro* degradation profiles of Rod-CC NPs in phosphate-buffered saline (PBS) with different pH values.

The *in vitro* degradation experiment was conducted under pH 5.6 and 7.4, and it aimed to stimulate the *in vivo* condition in the lysosome and body fluid [27]. Results in Figure 5 demonstrate the Rod-CC NPs degraded more than 45% under pH 5.6 and more than 20% under pH 7.4 in three months with a rapid degradation in the first week and a linear degradation in the last several weeks. The good degradation property of Rod-CC NPs ensures its biosafety and degradation is the key issue that affects the behavior of osteoblast cells.

### 2.2. Cytocompatibility

**Figure 6:**
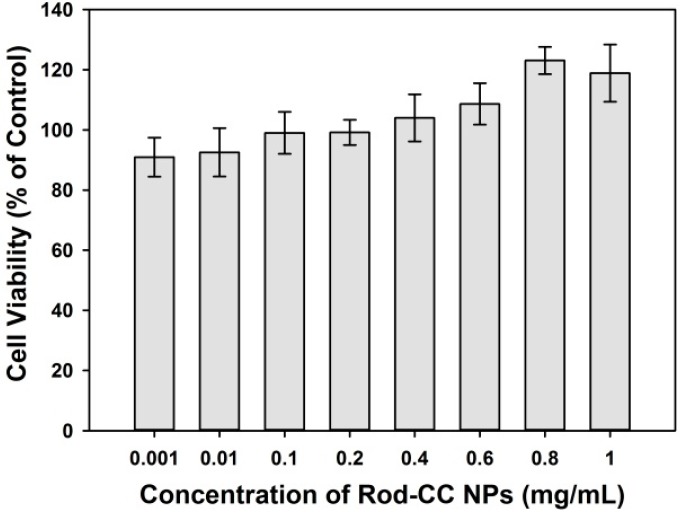
Cell viabilities of MC3T3-E1 cells treated with different concentrations of Rod-CC NPs for 48 h.

The cytocompatibility assay was conducted to assess whether the Rod-CC NPs were biocompatible enough to be used in a biological system. The cytocompatibility assay was carried out using Rod-CC NPs at concentrations of 0.001–1 mg/mL. The cell viabilities of all the groups were more than 80% when compared with the control, indicating that the Rod-CC NPs were biocompatible with osteoblast cells (Figure 6). Calcium carbonate is one of the ingredients of natural bone [28,29], and PAA is proved to be nontoxic, which may lead to its good biocompatibility [30,31]. Especially, with the increase of the concentration of Rod-CC NPs, it tended to promote the growth of MC3T3-E1 cells which may be because of the degradation of Rod-CC NPs and the released calcium ions.

### 2.3. Cell Proliferation Assay

**Figure 7:**
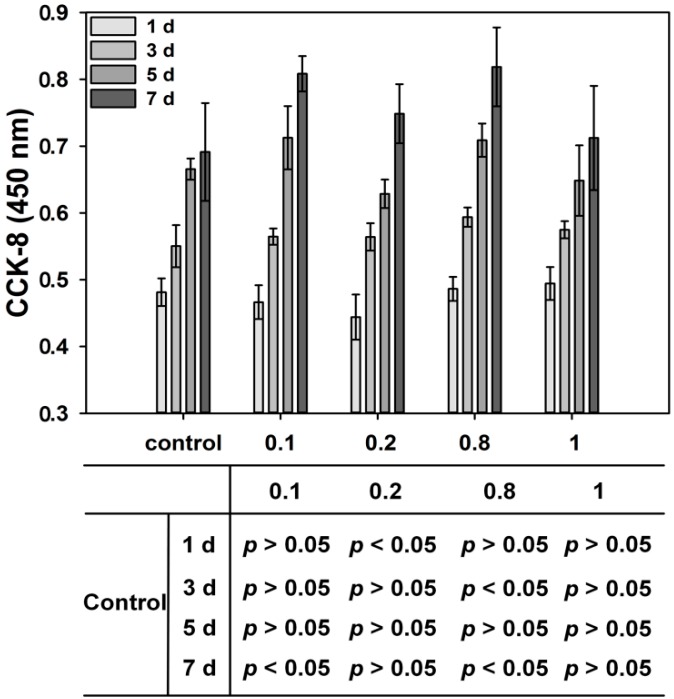
Cell proliferation of MC3T3-E1 cells cultured with different concentrations of Rod-CC NPs for one, three, five, and seven days. Table shows pair-wise comparison results of each group analyzed by Student-Newman–Keuls *post hoc* comparison test.

The CCK-8 assay was employed to measure the effects of Rod-CC NPs on the proliferation activity of MC3T3-E1 cells at day 1, 3, 5, and 7 (Figure 7). For control, the cell viability continuously increased within five days, and possibly due to the confluence among cells, the proliferation became relatively stable after day 5. However, with 0.1, 0.2, 0.8 and 1 mg/mL groups, the cell viability gradually increased within seven days.

The 0.1 and 0.8 mg/mL Rod-CC NP groups had a significant positive effect on the proliferation of MC3T3-E1 cells compared with the control (*p* < 0.05). For day 1, the 0.2 mg/mL group showed a significantly lower proliferation value than the control (*p* < 0.05) which may be related to the cell condition, and there was no significant difference between the 0.1, 0.8 and 1 mg/mL groups and the control (*p* > 0.05). At day 3, 0.8 mg/mL Rod-CC NPs had a higher proliferation value compared with the control (*p* < 0.05), and no statistical difference was found between the other groups and the control (*p* > 0.05). At day 5, no significant difference was found between the nanoparticle groups and the control (*p* > 0.05). At day 7, only the 0.1 and 0.8 mg/mL Rod-CC NPs groups exhibited higher proliferation values than the control (*p* < 0.05). The results depicted that the Rod-CC NPs had a significant positive effect on the proliferation of MC3T3-E1 cells at the concentrations of 0.1 and 0.8 mg/mL. Additionally, this may be ascribed to the appropriate calcium ion release, the penetration and influence on the Ca^2+^ signal, or the related proteins, which needs further mechanism research [32].

### 2.4. Differentiation of Osteoblast Cells

Based on the cell viability and proliferation studies, Rod-CC NP concentrations of 0.1 and 0.8 mg/mL were chosen to study the osteogenic differentiation of MC3T3-E1 cells.

**Figure 8:**
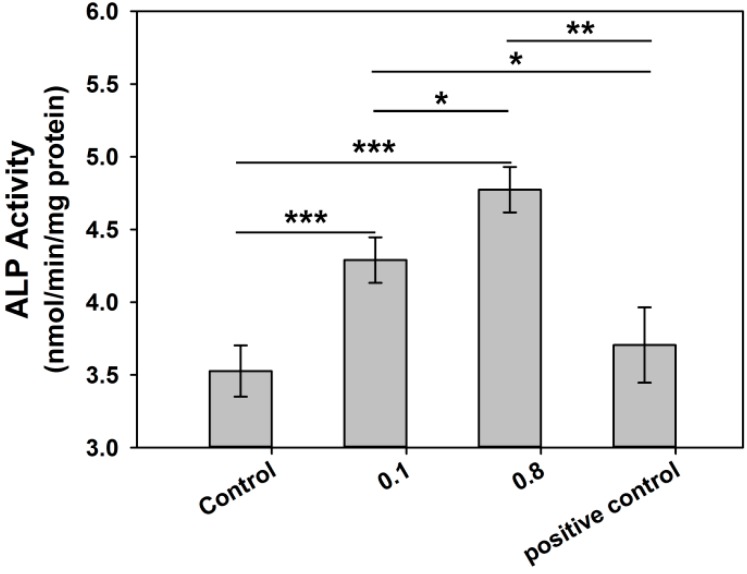
ALP activities of MC3T3-E1 cultured with different concentrations of Rod-CC NPs, and the osteoinduced medium without nanoparticles was employed as positive control. (* *p* < 0.05, ** *p* < 0.01, *** *p* < 0.001; *n* = 5).

For early the osteogenic differentiation test, the Alkaline phosphatase (ALP) assay was conducted (Figure 8). The two different concentrations of Rod-CC NPs were added to the seeded cells in growth medium, and the osteoinduced medium without nanoparticles was used as a positive control. As for the 0.1 mg/mL group, the ALP value was higher than the control (*p* < 0.001) and the positive control (*p* < 0.05). The Rod-CC NPs of 0.8 mg/mL also had better ALP activity than the positive control (*p* < 0.01) and the control (*p* < 0.001). Moreover, the 0.8 mg/mL group displayed significantly better improvement of ALP activity than the 0.1 mg/mL group.

**Figure 9:**
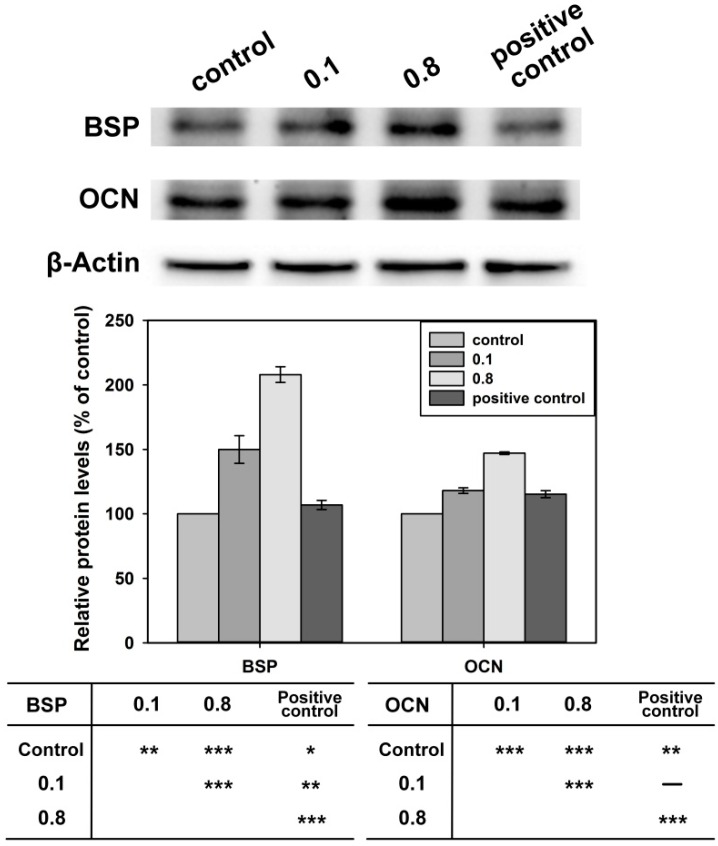
Western blot assays of the bone sialoprotein (BSP), osteocalcin (OCN) and β-Actin expression of MC3T3-E1 cells when combined with 0.1 and 0.8 mg/mL Rod-CC NPs, and the osteoinduced medium without nanoparticles was used as positive control. Table shows pair-wise comparison results of each group (* *p* < 0.05, ** *p* < 0.01, *** *p* < 0.001; *n* = 4).

Osteocalcin (OCN) and bone sialoprotein (BSP) are symbols of the advanced stage of osteogenic differentiation [33,34]. Therefore, the Western blot assay was conducted to evaluate the expression of OCN, BSP and β-Actin after the cells were cultured with 0.1 and 0.8 mg/mL Rod-CC NPs, and the osteoinduced medium without nanoparticles was used as a positive control. As shown in Figure 9, the expressions of BSP and OCN cultured with 0.1 and 0.8 mg/mL Rod-CC NPs were significantly higher than the control (*p* < 0.01) which indicated the nanoparticles enhanced the differentiation of MC3T3-E1 cells. Compared with the positive control, BSP expressions were significantly enhanced (*p* < 0.01) in both 0.1 and 0.8 mg/mL Rod-CC NP groups, and OCN expressions were comparable in the 0.1 mg/mL group (*p* > 0.05) and higher in the 0.8 mg/mL group (*p* < 0.001). These results illustrated that the Rod-CC NPs had the capacity to induce the differentiation of MC3T3-E1 cells.

Various parameters comprehensively regulate the processes of osteoblastic proliferation and differentiation, including hormonal regulation, physical stimulation and extracellular matrix maturation [33,35]. The calcium ion, which is considered to a coupling factor between osteoblasts and osteoclasts, plays a significant role in regulating the proliferation and differentiation of osteoblast cells by affecting the expression of calcium-dependent protein and specific Ca^2+^ channel isoforms [36]. According to Parakhonskiy, elongated particles (with a higher aspect ratio) have a higher internalized rate, and hence the synthesized Rod-CC NPs are supposed to have high internalized possibility [22]. After entering cells, the Rod-CC NPs with a rough surface begin to react with enzymes such as carbonic anhydrase and then they degrade and release Ca^2+^, and the variation of Ca^2+^ affects the Ca^2+^-mediated cellular responses to finally induce the proliferation and differentiation in due time [37]. These results are in accordance with the observation by Maeno [35].

Based on the biodegradability and osteoinduced ability, the Rod-CC NPs are able to be formed into bone-filling or bone substitute materials. Moreover, with the rough surface, Rod-CC NPs are supposed to have the capacity to load drugs or bioactive molecules which may obtain better bone regenerative effects.

## 3. Materials and Methods

### 3.1. Preparation of Rod-CC NPs

The pure Rod-CC NPs were synthesized with the regulation of PAA [38]. First, PAA (*M*_W_ = 5000, Acros Organics, Belgium, NJ, USA) was dissolved at a final concentration of 2 mM with deionized water and removed 10 mL to a round bottomed flask. The pH value was adjusted to 11 by 5 mM NaOH solution. Next, 7 mL of a 0.1 M CaCl_2_ aqueous solution (adjusted to pH 8.5 with NH_3_·H_2_O) was added dropwise to the prepared PAA solution at a rate of 1 mL/min under gentle stirring in water bath at 60 °C. After stirring for 60 min at 60 °C, 7 mL of a 0.1 M (NH_4_)_2_CO_3_ aqueous solution (adjusted to pH 10 with NH_3_·H_2_O) was added dropwise to the reaction solution under same condition. After keeping stirring for 24 h in the water bath, the calcium carbonate nanoparticles were ultra-centrifuged at 10,000 rpm for 5 min and washed with deionized water and ethanol each three times. Then the obtained products were dried at 60 °C in oven for 24 h and stored at 37 °C for further use.

### 3.2. Characterization of the Rod-CC NPs

#### 3.2.1. Physical–Chemical Characterization

The surface morphology, shape and size of the prepared nanoparticles were observed by scanning electron microscope (SEM, Hitachi S-4800, Tokyo, Japan). Fourier transform infrared spectroscopy (FTIR, Nicolet 5700, Thermo Electron, Waltham, MA, USA), thermogravimetric analysis (SDT Q600, TA Instruments, New Castle, PA, USA), X-ray diffraction (XRD, Rigaku Corporation, Tokyo, Japan) and Zeta Sizer Nano Series ZEN 3600 Spectrometer (Malvern Instruments Ltd., Malvern, Worcestershire, UK) were also employed to characterize the nanoparticles.

#### 3.2.2. Aqueous Stability of the Rod-CC NPs

Rod-CC NPs were dispersed in deionized water at a final concentration of 1 mg/mL, and placed in a cuvette for 3, 6, 9, 12 h and 1, 3, 5, 7 day at room temperature without moving and a picture was taken at each time point. The water dispersibility and stability of the nanoparticles could be estimated based on the precipitation conditions and the transparency of the solution.

#### 3.2.3. *In Vitro* Rod-CC NPs Degradation

Rod-CC NPs (50 mg) were immersed in 10 mL PBS solution at pH 5.6 and 10 mL PBS at pH 7.4, respectively. Then the solutions were gently stirred at a shaking bath at 37 °C for three months. At specific times, the supernatants were collected through centrifugation and same volume of fresh PBS solutions were added. QuantiChrom™ Calcium Assay Kit (BioAssay Systems, Hayward, CA, USA) was used to measure calcium concentration in the supernatant according to instructions. The final degradation percentage was calculated according to the released Ca^2+^ concentration tested above.

### 3.3. Cell Culture

MC3T3-E1 cells (Shanghai Cell Collection, Shanghai, China) were cultured in growth medium (GM) consisting of alpha-minimum essential medium (α-MEM) media with 10% FBS. For osteogenic induction, cells were cultured in osteoinduced medium (OM) which was obtained by adding 10 mM β-glycerophosphate, 50 μg/mL ascorbic acid and 10^−8^ M dexamethasone into GM. The cells were maintained in the density range of (0.1–1) × 10^6^ cells/mL. And all the cells were cultured in a humidified incubator at 37 °C and 5% CO_2_ atmosphere.

### 3.4. Cytocompatibility Study

To determine the cytocompatibility of the synthesized Rod-CC NPs, Cell Counting Kit-8 assay (CCK-8, Beyotime Institute of Biotechnology, Haimen, China) was performed. Then 100 μL MC3T3-E1 cells were seeded in 96-well plates at a concentration of 5 × 10^3^ cells/well. The cells were cultured with 20 μL of prepared Rod-CC NPs solution at the concentrations of 0.001, 0.01, 0.1, 0.2, 0.4, 0.6, 0.8 and 1 mg/mL. Cells cultured without nanoparticles were used as control. After being incubated for 48 h, CCK-8 assays were conducted according to the instructions. Briefly, after changing with 100 μL new growth medium, 10 μL CCK-8 solution was added to each well. Optical density (OD) values were detected at 450 nm after incubation at 37 °C for 2 h. Each sample has six parallel replicates. Cell viability was calculated as percentage of nanoparticles-cultured samples to control.

### 3.5. Cell Proliferation Study

Based on the cytocompatibility results, Rod-CC NPs at concentrations of 0.1, 0.2, 0.8 and 1 mg/mL were chose for the proliferation study. In detail, after seeded in 96-well plates, the cells were treated with 20 μL of 0.1, 0.2, 0.8 and 1 mg/mL of the prepared nanoparticle solution. Cells cultured without nanoparticles were used as control. After being incubated for 1, 3, 5, 7 days, CCK-8 assays were performed to determine the cell viability based on the above instructions. Each sample has six parallel replicates.

### 3.6. Osteogenic Differentiation Study

#### 3.6.1. Alkaline Phosphatase (ALP) Activity

ALP activity was measured to evaluate the early osteoblast differentiation [39]. Cells were seeded into 96-well plates with 5 × 10^3^ cells/well. After 24 h, the medium was changed to new growth medium with Rod-CC NPs in it, and the osteoinduced medium was used as positive control. Cultured for seven more days, the medium was discarded and cells were rinsed by PBS, then LabAssay ALP kit (Wako Pure Chemical Industries, Ltd., Osaka, Japan) was used to measure the ALP activity. RIPA buffer was used for protein extraction and BCA Protein Assay Reagent (Thermo Fisher Scientific, Waltham, MA, USA) was employed to determine the protein concentration. ALP activity was calculated as nmol of *p*-nitrophenol formation/min/mg of total proteins.

#### 3.6.2. Osteocalcin and Bone Sialoprotein Production

To further demonstrate osteogenic differentiation, bone sialoprotein (BSP) and osteocalcin (OCN) synthesis were measured by Western blot. After 21 days cultured with Rod-CC NPs at 6-well plates, MC3T3-E1 cells were carefully washed by rinsing ice-cold PBS and lysed using Cell lysis buffer for Western and IP on ice for 30 min. After being centrifuged at 12,000 rpm for 10 min at 4 °C, the protein concentrations were measured by bicinchoninic acid (BCA) assay. For Western blot analysis, 20 μg of each protein sample was used for sodium dodecyl-sulfate polyacrylamide gel electrophoresis (SDS-PAGE) and then was electrotransferred onto poly(vinylidene fluoride) (PVDF) membranes. After being blocked with 5% bovine serum albumin for 2 h, the membranes were incubated with 1:1000 diluted primary anti-osteocalcin antibody (Sigma-Aldrich, St. Louis, MO, USA), anti-bone sialoprotein antibody (Abcam, Cambridge, UK) and β-actin antibody (Beyotime Institute of Biotechnology, Haimen, China) respectively at 4 °C overnight and then with 1:2000 diluted secondary antibody for 1 h at room temperature. Signals were determined using the chemiluminescence image analysis system (Tanon 5500, Shanghai, China).

### 3.7. Statistical Analysis

Significance Tests were conducted using Student’s *t*-test. A *p*-value less than 0.05 was considered statistically significant. Pairwise comparisons were conducted with the Student-Newman–Keuls *post hoc* comparison test.

## 4. Conclusions

Rod-like calcium carbonate nanoparticles with desired water dispersibility were successfully synthesized. The prepared Rod-CC NPs had an average length of 240 nm, a width of 90 nm with an average aspect ratio of 2.60 and a negative ζ-potential of −22.25 ± 0.35 mV. Within three months, the Rod-CC NPs degraded 23% and 45% in PBS solution at pH 7.4 and 5.6, respectively. The *in vitro* study demonstrated the Rod-CC NPs at concentrations of 0.1 and 0.8 mg/mL had the capacity to improve the proliferation and induce the differentiation of MC3T3-E1 cells.
